# JaponiconeA induces apoptosis of bortezomib-sensitive and -resistant myeloma cells *in vitro* and *in vivo* by targeting IKKβ

**DOI:** 10.20892/j.issn.2095-3941.2020.0473

**Published:** 2021-09-28

**Authors:** Zilu Zhang, Chenjing Ye, Jia Liu, Wenbin Xu, Chao Wu, Qing Yu, Xiaoguang Xu, Xinyi Zeng, Huizi Jin, Yingli Wu, Hua Yan

**Affiliations:** 1 Shanghai Institute of Hematology, Affiliated Ruijin Hospital of Shanghai Jiao Tong University School of Medicine, Shanghai 200025, China; 2 VIP Health Center, Affiliated Ruijin Hospital of Shanghai Jiao Tong University School of Medicine, Shanghai 200025, China; 3 Shanghai Key Laboratory for Molecular Engineering of Chiral Drugs, School of Pharmacy, Shanghai Jiao Tong University, Shanghai 200240, China; 4 Hongqiao International Institute of Medicine, Shanghai Tongren Hospital/Faculty of Basic Medicine, Chemical Biology Division of Shanghai Universities E-Institutes, Key Laboratory of Cell Differentiation and Apoptosis of the Chinese Ministry of Education, Shanghai Jiao Tong University School of Medicine, Shanghai 200025, China

**Keywords:** Multiple myeloma, NF-κB, JaponiconeA, bortezomib, drug resistance

## Abstract

**Objective:**

Multiple myeloma (MM) remains incurable with high rates of relapse. New therapeutic drugs are therefore urgently needed to improve the prognosis. JaponiconeA (JA), a natural product isolated from *Inula japonica Thunb*, has shown good anti-MM potential. A comprehensive study should therefore be conducted to identify both the *in vitro* and *in vivo* mechanisms of the anti-MM effects of JA.

**Methods:**

CCK8 assays and flow cytometry were used to detect the proliferation, apoptosis, and cell cycle of MM cell lines when treated with JA. *In vivo* experiments were conducted using subcutaneous xenograft mouse models. We also identified possible targets and the mechanism of JA using RNA-seq and c-Map databases, and identified the specific targets of JA in bortezomib-sensitive and -resistant MM cell lines using CETSA, DARTS, and rescue experiments. Furthermore, JA and bortezomib were used separately or together to characterize their possible synergistic effects.

**Results:**

*In vitro*, JA inhibited proliferation, and induced apoptosis and G2/M phase arrest in MM cell lines, and selectively killed primary CD138^+^ MM cells. *In vivo*, JA also demonstrated a strong anti-tumor effect with no observable toxicity. In addition, JA showed synergetic effects in combination with bortezomib, and enhanced the anti-tumor effect of bortezomib in bortezomib-resistant cells. CETSA and DARTS confirmed direct binding of JA to NF-κB inhibitor kinase beta (IKKβ), and overexpression of IKKβ or knockdown of IκBα partially rescued the apoptosis induced by JA.

**Conclusions:**

JA exhibited strong anti-tumor effects in MM. It sensitized myeloma cells to bortezomib and overcame NF-κB-induced drug resistance by inhibiting IKKβ, providing a new treatment strategy for MM patients.

## Introduction

Multiple myeloma (MM) is the second most common hematological malignancy, characterized by the proliferation of clonal plasma cells in bone marrow, leading to symptoms including hypercalcemia, renal damage, anemia and bone diseases^1^. Despite the use of proteasome inhibitors and immunomodulatory drugs, MM is still an incurable disease, and almost all patients eventually relapse due to drug resistance^2^, so it is important to find new chemotherapeutic agents and strategies to improve the prognosis.

The nuclear factor-kappa B (NF-κB) signaling pathway has been reported to be activated in MM and contributes to its progression and drug resistance^3–5^. The activation of the NF-κB pathway induces expression of several anti-apoptotic proteins including Bcl-2, Bcl-XL, c-IAP1/2, c-FLIP, and XAIP^6–9^, which promote the survival of MM cells. Moreover, bortezomib treatment induces activation of the NF-κB pathway, which may limit the efficacy of bortezomib^10^, and a stronger activation was observed in bortezomib-resistant MM cells^11^. In addition, several studies have reported that activation of the NF-κB pathway was strongly associated with bortezomib resistance^12,13^. Thus, targeting the NF-κB pathway could be a possible strategy to treat MM and overcome bortezomib resistance.

Active natural compounds, isolated from traditional herbal medicines, are likely to be sources of new and effective anti-tumor drugs, which have minimal adverse effects^14,15^. JaponiconeA (JA) is a natural product isolated from *Inula japonica Thunb*^16^. A previous study showed that JA inhibited breast cancer cell proliferation by inhibiting the expression of RAD54B^17^. Moreover, JA inhibited the growth of non-small cell lung cancer cells *via* mitochondrial-mediated pathways^18^. JA is also an effective treatment for Burkitt lymphoma^19^. In the present study, we found that JA showed a potent anti-tumor effect for MM, both *in vitro* and *in vivo,* but showed no toxicity to normal cells, and showed enhanced cytotoxicity when combined with bortezomib. Further studies showed that JA targeted NF-κB inhibitor kinase beta (IKKβ) to inhibit the NF-κB pathway, and to induce cell apoptosis and cell cycle arrest. These results showed the promising preclinical performance of JA for the possible treatment of MM.

## Materials and methods

### Reagents and compounds

JA, with a purity of over 98%, was purified and kindly provided by Professor Huizi Jin from the Key Laboratory of Food Safety Research. Dimethyl sulfoxide (DMSO) was purchased from Sigma-Aldrich (St. Louis, MO, USA). Bortezomib and Cell Counting Kit-8 (CCK-8) were obtained from MedChemExpress (Monmouth Junction, NJ, USA). Antibodies to PARP-1, caspase3, caspase9, CDK1, CCNB1, β-actin, p65, p-p65, IKBα, and p-IKBα were purchased from Proteintech (Rosemont, IL, USA). The Cell IKKβ Fluo assay kit was purchased from Genmed (Cwmbran, UK).

### Cells culture methods

NCI-H929, OPM2, LP-1, RPMI 8226, and MM1.S myeloma cell lines were obtained from the American Type Culture Collection (Manassas, VA, USA) and were cultured and maintained in our own laboratory. NCI-H929 and MM1.S cells were cultured in RPMI-1640 medium, which was supplemented with 10% fetal bovine serum (FBS) and 100 IU/mL penicillin, and 100 μg/mL streptomycin. OPM2 and LP-1 cells were cultured in Iscove’s Modified Dulbecco’s Medium (IMDM) supplemented with 15% FBS, penicillin (100 IU/mL), and streptomycin (100 μg/mL). RPMI 8226 was cultured in IMDM with a higher FBS concentration (20%).

### Western blot

The whole cell lysates were extracted in 1× SDS, resolved using 8%–12% SDS-PAGE, and transferred to nitrocellulose membranes (Bio-Rad, Hercules, CA, USA). After blocking with 5% nonfat milk in phosphate-buffered saline, the membranes were incubated with antibodies overnight at 4 °C, followed by incubation in horseradish peroxidase (HRP)-linked secondary antibody (Cell Signaling Technology, Beverly, MA, USA) for 1 h at room temperature. The signals were detected using a chemiluminescence phototope-HRP kit (Cell Signaling Technology), according to the manufacturer’s instructions.

### Detection of apoptosis

Apoptosis was detected using an eBioscience™ Annexin V Apoptosis Detection Kit (Thermo Fisher Scientific, Waltham, MA, USA) according to the instructions from the manufacturer. Briefly, MM cells were treated with JA for specific times, and approximately 1 × 10^6^ cells were then harvested and washed once with 1× binding buffer and then resuspended in 100 μL of 1× loading buffer. Then, 5 μL propidium iodide (PI) and 5 μL Annexin V-APC were added per sample to the cell suspension, followed by incubation in the dark for 15 min. The apoptotic cells were quantified using a flow cytometer (Fortessa, San Francisco, CA, USA) using DIVA software. Approximately 10,000 cells were analyzed for each sample.

### Cell cycle assay

The distribution of the cell cycle was determined by measuring the DNA content using flow cytometry, as previously described^20^. In brief, MM cells were fixed with 75% ethanol for at least 12 h at −20 °C, and then incubated with 50 μg/mL RNase A and 100 μg/mL PI for 30 min, respectively. The DNA content was determined by a flow cytometer (Fortessa). The distribution of cells in the cell cycle was analyzed by Flowjo software. A total of 20,000 cells were gated and analyzed for each sample.

### Xenograft mouse model

The experimental protocol was approved by the Shanghai Jiao Tong University School of Medicine Institutional Animal Care & Use Committee (A-2015-008). Female BALB/c nu/nu mice (5–6 weeks of age) were purchased from Beijing Vital River Laboratory Animal Technology (Beijing, China) and kept in specific pathogen-free conditions at the Animal Center of Ruijin Hospital. A human myeloma xenograft model was established by subcutaneously inoculating 1 × 10^7^ NCI-H929/H929-BR cells into the region near the armpits of the forelimbs of mice. When tumor masses were visible, the mice were randomly divided into the JA and control groups to receive treatments. JA was intraperitoneally administrated at 30 mg/kg once a day for 10 days. The body weight of the mice and length (L) and width (W) of tumors were monitored every day, beginning with the first treatment. Tumor growth was evaluated by measuring the tumor volume using the formula: (V) = L × W^2^/2. After 10 treatments, the mice were sacrificed and tumor masses were removed and photographed.

### Cellular thermal shift assay (CETSA)

MM cell lines were harvested and diluted in RIPA supplemented with protease inhibitor cocktail and phenylmethanesulfonyl fluoride. The cell suspensions were freeze-thawed 3 times in liquid nitrogen. The soluble fraction was then separated from the cell debris and divided into 2 aliquots. One aliquot was treated with DMSO and the other aliquot with JA. After a 30 min incubation at 37 °C, the respective lysates were divided into separate aliquots (30 μL), and Western blot was used to analyze their contents.

### Primary MM cells and bone marrow (BM) mononuclear cells

Patients and healthy volunteers signed the informed consent forms before sample collections. The study was conducted in accordance with the Declaration of Helsinki protocol. It was approved by the Ethics Committee of the Affiliated Ruijin Hospital of Shanghai Jiao Tong University School of Medicine (2020-No.403). BM mononuclear cells were isolated from using Ficoll-Hypaque density gradient sedimentation (Pharmacia, Piscataway, NJ, USA). CD138^+^ myeloma cells of active MM patients were obtained using EasyStep CD138^+^ microbeads (Stem Cell Technologies, Vancouver, Canada).

### Immunofluorescence analysis

Cells were fixed with 4% paraformaldehyde and treated with 0.3% Triton X-100, then blocked with bovine serum albumin. The cells were then incubated with antibody against p65 overnight at 4 °C, followed by treatment with fluorescein isothiocyanate-labeled anti-rabbit immunoglobulin G antibody (Invitrogen, Carlsbad, CA, USA) and 4′,6-diamidino-2-phenylindole. The stained cells were examined with a fluorescence microscope (Nikon, Tokyo, Japan).

### Immunohistochemical analysis

The tumors were fixed with formalin, paraffin embedded, then sectioned into slices. The tissue sections were stained with hematoxylin and eosin, then used for the TUNEL and anti-Ki67 immunoassays.

### Drug affinity responsive target stability (DARTS)

DARTS, a fast and robust method to determine direct binding of a small molecule without requiring large amounts of pure protein, was performed as previously described^21–23^. MM cells (2–3 × 10^7^) were lysed for 10 min using M-PER supplemented with protease and phosphatase inhibitors. The supernatants were then treated with JA or the DMSO control for 30 min and centrifuged at 14,000 rpm for 15 min. After drug incubation, the proteins were digested with pronase for 30 min. The protein digestion was terminated by adding 4× sample buffer and heating the samples to 95 °C for 5 min.

### The in vitro IKK kinase activity assay

A IKKβ kinase activity quantitative detection kit (Genmed) was used to evaluate the JA-mediated inhibition of IKKβ kinase activity *in vitro.* In brief, MM cells were lysed with the lysis buffer in the kit, and a microplate reader was used to measure the total and nonspecific activities. The data were analyzed to determine the specific activity by using a formula given in the detection kit manual.

### Plasmid IKK overexpression

The IKKβ overexpression plasmid was purchased from the DNA library of Shanghai Jiao Tong University School of Medicine (Shanghai, China). The OE-IKKβ or vector plasmid were transfected with the lentivirus packaging vectors, psPAX2 and pMD2.G, introduced into HEK293T cells to produce lentivirus. The lentivirus was harvested to infect the MM cells.

### Real-time fluorescence quantitative PCR (qPCR)

Real-time fluorescence quantitative PCR (qPCR) was performed with a TransStart^®^ Top Green qPCR SuperMix Kit (Transgen, Beijing, China), and the primers listed below were synthesized by Sangon Biotech (Shanghai, China).

Bax forward: 5′-TCAGGATGCGTCCACCAAGAAG-3′;

  reverse: 5′-TGTGTCCACGGCGGCAATCATC-3′;

Bcl-xl forward: 5′-GCCACTTACCTGAATGACCACC-3′;

  reverse: 5′-AACCAGCGGTTGAAGCGTTCCT-3′;

c-Myc forward: 5′-CCTGGTGCTCCATGAGGAGAC-3′;

  reverse: 5′-CAGACTCTGACCTTTTGCCAGG-3′;

ICAM1 forward: 5′-AGCGGCTGACGTGTGCAGTAAT-3′;

  reverse: 5′-TCTGAGACCTCTGGCTTCGTCA-3′;

IKKβ forward: 5′-ACAGCGAGCAAACCGAGTTTGG-3′;

  reverse: 5′-CCTCTGTAAGTCCACAATGTCGG-3′.

### Statistical analysis

The statistical significance of differences observed in drug-treated versus control cultures was determined using the Wilcoxon signed-rank test. The minimal level of significance was a value of *P* < 0.05.

## Results

### JA inhibited proliferation and induced cell cycle arrest, which led to apoptosis of MM cells

**Figure 1:**
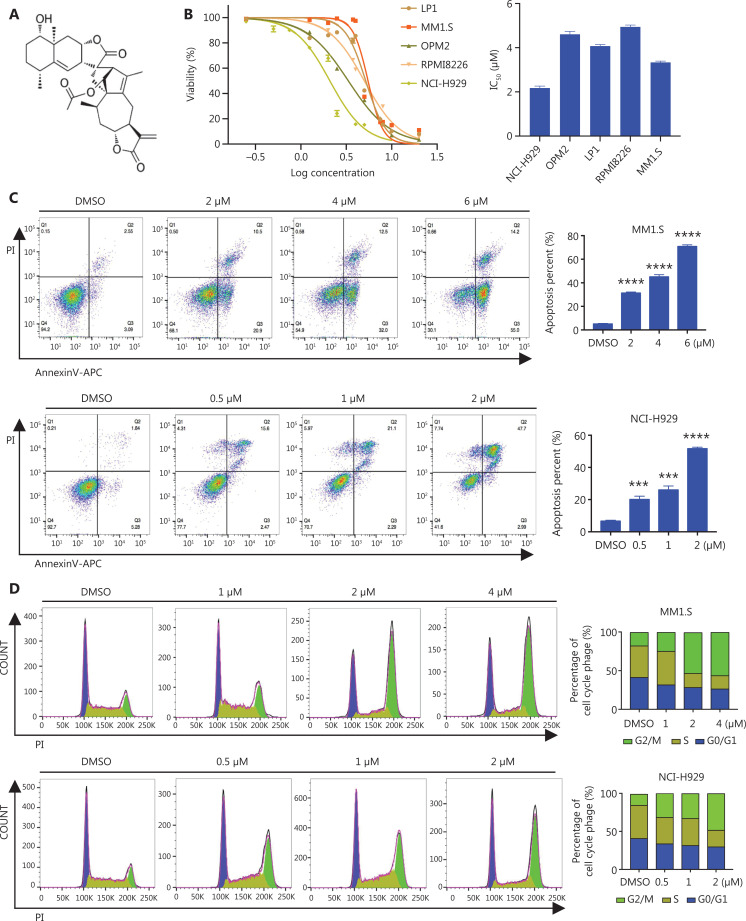

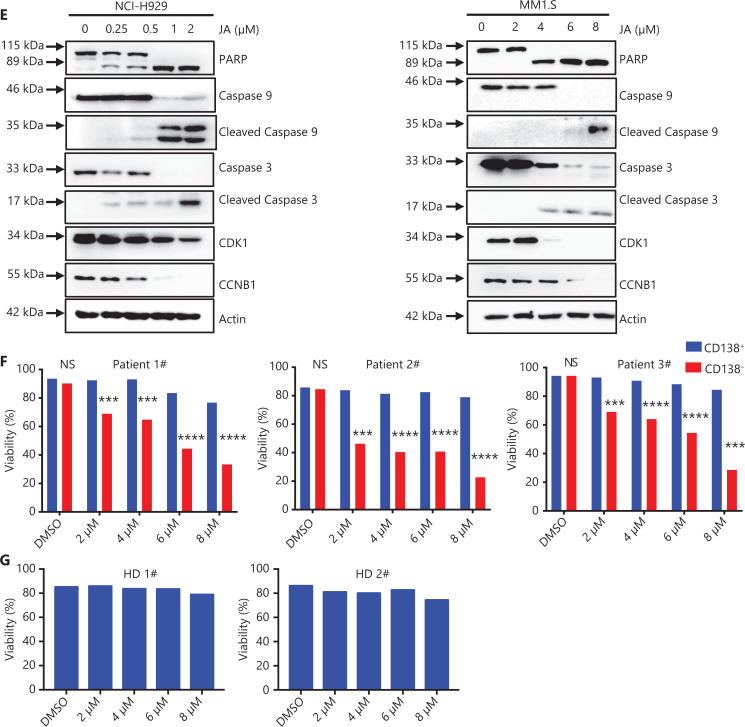
JaponiconeA (JA) inhibited proliferation, induced cell cycle arrest, leading to apoptosis in MM cells. (A) The chemical structure of JA. (B) The inhibition of JA on MM cell lines for 24 h was detected with the CCK8 assay, and the IC_50_ was calculated using Graphpad prism software. (C) (left) MM cells were exposed to various concentrations of JA for 24 h, and apoptotic cells were analyzed by flow cytometry (right). The data shown are the percentages of apoptotic cells from at least 3 independent experiments with similar results. (D) MM cells were treated with JA for 24 h, and the cell cycle was analyzed by flow cytometry. (E) MM cells were treated with the indicated concentrations of JA for 24 h, followed by Western blot to detect the indicated proteins. (F) CD138^+^ myeloma cells and CD138^-^ cells isolated from MM patients were treated with the indicated concentrations of JA, and the cell viability was determined after 48 h. (G) Healthy donor bone marrow mononuclear cells were treated with JA for 48 h and the cell viability was measured using the CCK-8 assay (*P < 0.05; **P < 0.01; ***P < 0.001 *vs.* the control).

JA was extracted from the traditional herbal medicine, *Inula japonica Thunb*. The chemical structure of JA is shown in **Figure 1A**. The anti-tumor effects of other *Inula* sesquiterpenoids have been extensively studied^24^. To further assess the potential anti-tumor effect of JA, we determined the effects of JA in a variety of hematological malignant cell lines and found that myeloma cells were highly sensitive to JA, with average half-maximal inhibitory concentration (IC_50_) ranging from 2.2 μM to 4.8 μM (**Figure 1B**). To determine the effect of JA on apoptosis in MM cells, MM1.S and NCI-H929 cells were exposed to different concentrations of JA for 24 h, showing that JA induced MM cell apoptosis in a dose-dependent manner (**Figure 1C**). Moreover, apoptosis-related proteins also showed corresponding changes (**Figure 1E**). The effect of JA on cell cycle distribution was also determined using flow cytometry. Exposure to JA increased the percentage of cells in the G2/M phase, indicating that JA caused G2/M phase cell cycle arrest in MM cells (**Figure 1D**). The G2/M-related cell cycle checkpoint proteins were also detected (**Figure 1E**). Primary myeloma cells from MM patients and BM mononuclear cells from healthy donors were then exposed to JA treatment for 48 h. JA selectively killed CD138^+^ myeloma cells while sparing the normal cells (**Figure 1F, G**). Together, these results showed that JA had a potent anti-tumor effect on myeloma cells, with much less toxicity of normal cells, highlighting its therapeutic potential in treating MM patients.

### JA inhibited MM cell proliferation in a xenograft model

**Figure 2:**
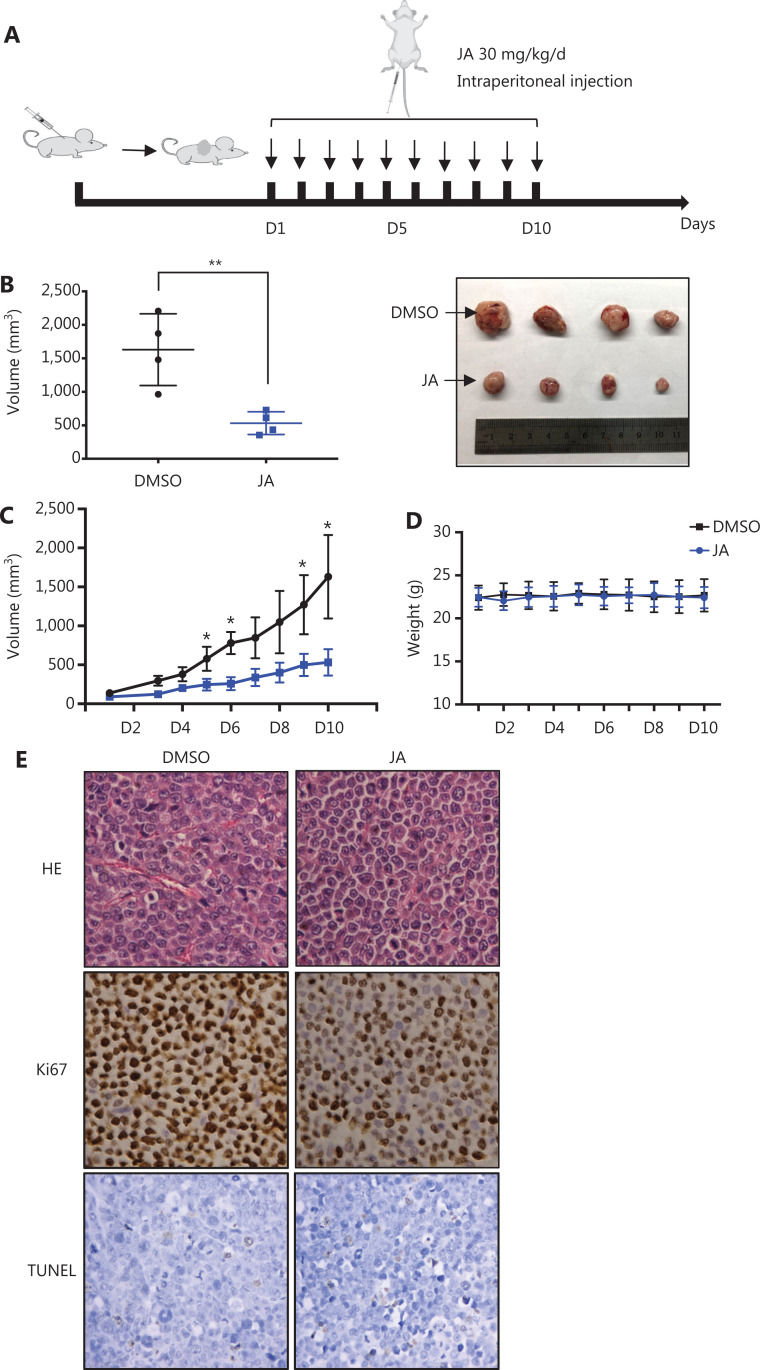
JaponiconeA (JA) inhibited MM tumor growth in a xenograft mice model. (A) Schematic diagram of the *in vivo* experiments. (B, C) JA (30 mg/kg) was intraperitoneally administrated daily for 10 days, and the tumor volumes were measured each day during the treatment period. On day 10, mice were sacrificed. Tumors were removed and the tumors of each group were placed together for the photographs. (D) The body weight of mice was recorded everyday 2 days during the treatment period. (E) Ki67 and TUNEL were detected using immunohistochemical analyses of the xenograft tumors in each group (200 x, *P < 0.05; **P < 0.01 *vs*. the control).

The anti-tumor activity of JA was also investigated *in vivo*. A myeloma xenograft model was established as described in the Materials and Methods. The exact procedures are summarized in a schematic (**Figure 2A**). JA treatment significantly reduced the tumor burden, when compared with the control group (**Figure 2B and 2C**). Mice in the JA treatment group showed no significant weight loss or other signs of toxicity during the administration (**Figure 2D**). The representative pathological images of the tumors in both the JA treatment and control groups are shown in **Figure 2E**. The percentage of Ki67-positive cells was significantly decreased and the TUNEL signal was slightly enhanced after treatment with JA, indicating that JA strongly inhibited the growth of MM cells and partially induced apoptosis in MM cells *in vivo*. These results further showed that JA had an excellent anti-tumor effect.

### JA potentiated the cytotoxic effect of bortezomib and partially overcame drug resistance to bortezomib

**Figure 3:**
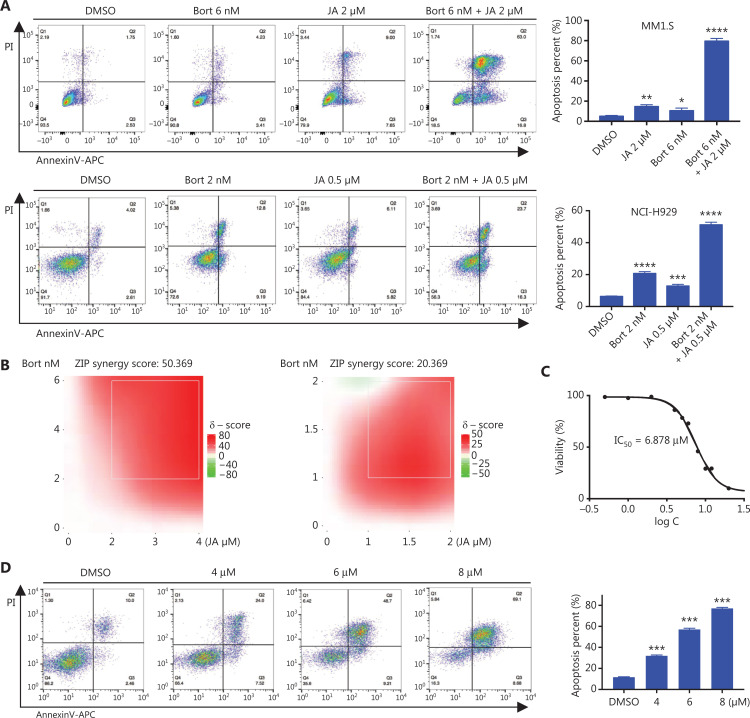

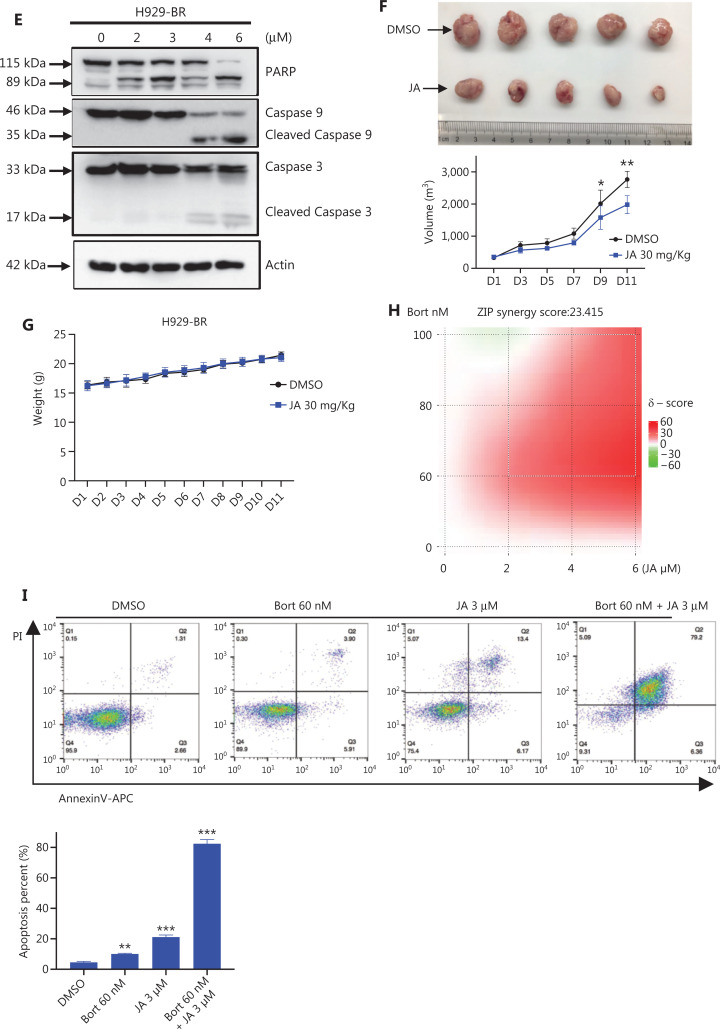
JaponiconeA (JA) potentiated the cytotoxic effect of bortezomib and partially overcame drug resistance to bortezomib. (A) MM cells were treated with bortezomib, JA separately, or together for 24 h, and the number of apoptotic cells was determined using flow cytometry with Annexin V/propidium iodide (PI). (B) MM cells were treated with bortezomib, JA separately, or together for 24 h, and the inhibition was determined using the CCK8 assay. The combination index (CI) was then analyzed using CompuSyn software. (C) H929-BR or NCI-H929 cells were treated with bortezomib for 24 h, and the IC_50_ was calculated using Graphpad prism software. (D) H929-BR cells were treated with JA for 24 h, and the IC_50_ was calculated using Graphpad prism software. (E) H929-BR cells were treated with JA for 24 h and apoptotic cells were detected by flow cytometry with Annexin V/PI. (F) JA (30 mg/kg) was intraperitoneally administrated daily for 11 days, and tumor volumes were measured. On day 11, the mice were sacrificed. Tumors were removed and tumors of each group were placed together and photographed. (G) The body weights of mice were recorded every 2 days during the treatment period. (H) H929-BR cells were treated with bortezomib/JA for 24 h to calculate the CI of JA and bortezomib. (I) After exposure to JA/bortezomib for 24 h, the numbers of apoptotic cells were determined by flow cytometry with Annexin V/PI (*P < 0.05; **P < 0.01; ***P < 0.001; ****P < 0.0001 *vs.* the control).

Bortezomib is a first-line therapeutic agent and has significantly improved the prognosis of MM patients, but most patients eventually relapse with drug resistance. Considering the strong anti-MM effects of JA, we assessed whether JA and bortezomib had synergistic effects in their anti-myeloma activities. MM cells were treated using JA and bortezomib separately, or together for 24 h, followed by detection of apoptosis. The percentage of apoptotic cells increased significantly in the combination treatment group (**Figure 3A**). The combination index (CI) of JA and bortezomib was also calculated, further demonstrating the synergistic effect of JA and bortezomib (**Figure 3B, Supplementary Figure S1, Table 1**). 

**Table 1:** The combination index (*CI*) of JaponiconeA and bortezomib in MM1.S cells

Bort (nM)	JA (μM)	Effect	*CI*
6	4	0.83296	0.35718
6	3	0.76542	0.32648
6	2	0.69791	0.27206
4	4	0.7939	0.38506
4	3	0.69771	0.3618
4	2	0.32335	0.59297
2	4	0.63334	0.50302
2	3	0.55884	0.43409
2	2	0.17082	0.78298
The combination index (*CI*) of JaponiconeA and bortezomib in H929 cells			
**Bort (nM)**	**JA (μM)**	**Effect**	***CI***
1	1	0.69	0.52271
1	1.5	0.9	0.31826
1	2	0.96	0.5498
1.5	1	0.56	0.92831
1.5	1.5	0.8	0.61115
1.5	2	0.92	0.40547
2.0	1	0.53	1.44047
2.0	1.5	0.765	0.92400
2.0	2	0.939	0.43554

We also verified the anti-MM activity of JA in bortezomib-resistant MM cells. Bortezomib-resistant H929 (H929-BR) cells were obtained after a long period of exposure to increasing amounts of bortezomib, with the tolerance being > 40 nM bortezomib, whose sensitivity to bortezomib was more than 10-fold lower than that of H929 cells (**Supplementary Figure S2**). Surprisingly, we found that JA was also effective in H929-BR cells, with a IC_50_ approximately 6.878 μM (**Figure 3C**). JA induced apoptosis in H929-BR cells in a dose-dependent manner, which was detected using both flow cytometry and Western blot (**Figure 3D and 3E**). The anti-tumor activity was also investigated *in vivo*. The myeloma xenograft model was established as previously mentioned. JA significantly reduced tumor burden, when compared with the DMSO group (**Figure 3F**), without weight loss or other signs of toxicity (**Figure 3G**). The synergistic effect of JA and bortezomib was also calculated in H929-BR cells. **Figure 3H and Supplementary Figure S3** (**Table 2**) show that strong synergism was observed between JA and bortezomib. Treatment with JA and bortezomib showed stronger apoptosis in H929-BR cells than single reagent treatment (**Figure 3I**).

**Table 2:** The combination index (*CI*) of JaponiconeA and bortezomib in H929-BR cells

Bort (nM)	JA (μM)	Effect	*CI*
100	6	0.82442	0.59389
100	4	0.69461	0.78560
100	2	0.52836	1.03257
80	6	0.80481	0.49955
80	4	0.67659	0.64910
80	2	0.50620	0.85486
60	6	0.7853	0.39232
60	4	0.62766	0.52914
60	2	0.49229	0.65508

### JA inhibited activation of the NF-κB pathway

**Figure 4:**
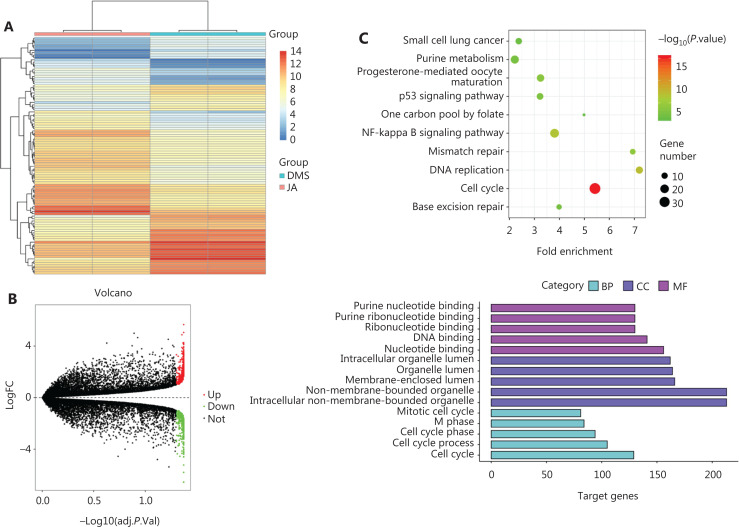

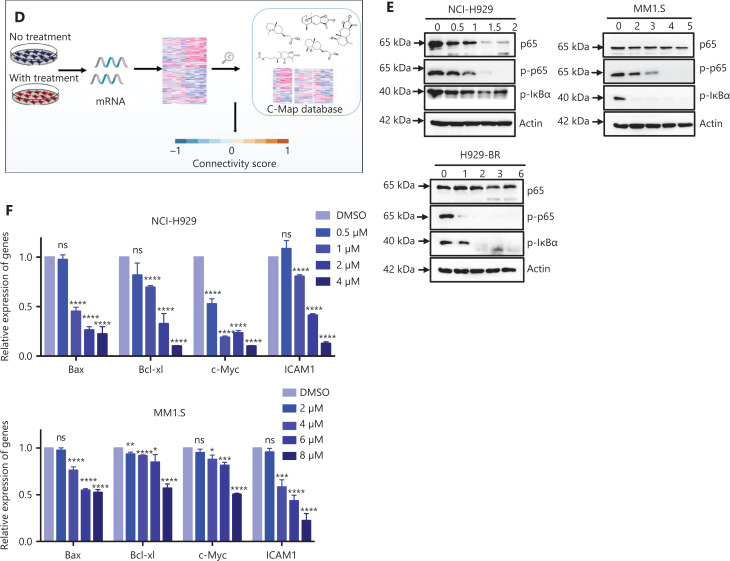
JaponiconeA (JA) inhibited the activation of the NF-κB pathway. (A) MM1.S cells were treated with JA or dimethyl sulfoxide for 24 h, and then the cells were harvested and subjected to next-generation sequencing. A clustering heat map was shown as above. (B) A volcano map was used to indicate differentially expressed genes (DEGs). A total of 1,001 DEGs were selected based on the following criteria: adjusted *P*-value < 0.05 and FC > 2. (C) GO and KEGG enrichment analyses was performed on downregulated DEGs. (D) A brief description of the mechanism of c-MAP. (E) The components of the NF-κB pathway of MM cells were detected after treatment with bortezomib for 24 h. (F) The mRNA levels of NF-κB target genes including BAX, BCL-xl, c-Myc, and ICAM1 were quantified using q-PCR after treatment with JA for 12 h (*P < 0.05; **P < 0.01; ***P < 0.001; ****P < 0.0001 *vs.* the control).

To further characterize the mechanism of the anti-tumor effect of JA on MM cells, MM1.S cells were treated with JA or DMSO for 24 h, then the cells were harvested and subjected to next-generation sequencing. A clustering heat map showed good separation of samples in the JA and DMSO groups (**Figure 4A**). A total of 1,001 differentially expressed genes (DEGs) were selected, including 498 upregulated genes and 503 downregulated genes, based on the criteria of an adjusted value of *P* < 0.05 and fold change > 2 (**Figure 4B**). Gene Ontology (GO) and Kyoto Encyclopedia of Genes and Genomes (KEGG) enrichment analyses were performed for the down-regulated DEGs, showing that the DEGs were predominately enriched in “signaling transduction” and cell cycle-associated molecular functions (**Figure 4C**). We found that the NF-κB pathway, an activated pathway contributing to the development and drug resistance of MM cells, was enriched in the KEGG annotation of down-regulated DEGs, suggesting that JA may have exerted its anti-MM effect by inhibiting the NF-κB pathway. The potential anti-tumor mechanism of JA was also analyzed using a connective map database (c-MAP). Using the c-MAP database, the expression characteristics of JA-treated cells were compared with those of cells treated with other compounds^25,26^. The compounds with similar expression signatures may share similar pharmacological mechanisms. A brief description of the operation of the database is shown in **Figure 4D**. Importantly, 3 of the top 10 predictions were NF-κB IKKβ inhibitors according to the c-MAP (**Table 3**). Additionally, we observed that phosphorylated p65 and IKBα were significantly downregulated in NCI-H929, MM1.S, and H929-BR cells, indicating strong inhibition of JA on the NF-κB pathway (**Figure 4E**). Also, the mRNA levels of NF-κB target genes were decreased after exposure to JA (**Figure 4F**). Together, these results demonstrated that JA inhibited the NF-κB pathway.

**Table 3:** The targets of small molecule compounds predicted by c-MAP

Targets	Drugs
IKKβ	Withaferin A Parthenolide Geldanamycin
HDAC1/2	Vorinostat Trichostatin A
HSP90	Geldanamycin
Wnt	Pyrvinium
m-TOR	Resveratrol

**Figure 5:**
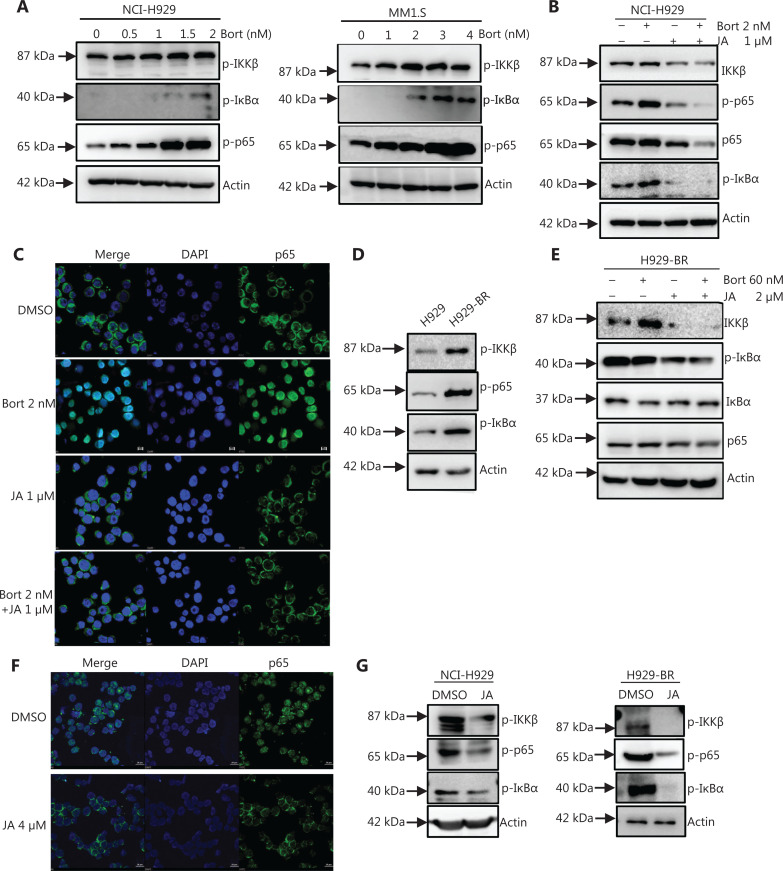
JaponiconeA (JA) inhibited the activation of the NF-κB pathway induced by bortezomib. (A) The components of the NF-κB pathway of MM cells were detected after treatment with bortezomib for 24 h. (B) NCI-H929 cells were treated by single or both drugs, followed by Western blot to determine the expressions of the indicated proteins. (C) NCI-H929 cells were treated with the indicated drugs for 24 h, and p65 was detected by immunofluorescence. (D) The expressions of p-IKKβ, p-p65, and p-IκBα in NCI-H929 and H929-BR cells were determined by Western blot. (E) H929-BR cells were treated by single or both drugs, followed by Western blot to detect specific proteins. (F) H929-BR cells were treated with JA for 24 h, and p65 was detected by immunofluorescence. (G) The detection of proteins of the NF-κB pathway in the NCI-H929 mouse tumors (left) and H929-BR mouse tumors (right) using Western blot.

It was reported that bortezomib induced the activation of the canonical NF-κB pathway in MM cells^10^. **Figure 5A** shows that the NF-κB pathway was indeed activated in both NCI-H929 and MM1.S cells after treatment with bortezomib. Treatment with JA strongly inhibited the activation of NF-κB induced by bortezomib (**Figure 5B and 5C**), which may help explain the synergistic effects of JA and bortezomib. In addition, the activity of NF-κB has been reported to be further enhanced in MM patients who were refractory to bortezomib therapy^11^. We therefore compared the activations of the NF-κB pathway in both H929-S and H929-BR cells. A stronger activation of this pathway was observed in H929-BR cells (**Figure 5D**). JA could also effectively downregulate the activation of NF-κB in H929-BR cells (**Figure 5E and 5F**). The anti-tumor effect of JA by the NF-kB signaling pathway was also validated *in vivo* (**Figure 5G**). Considering the important role of NF-κB activation in both sensitive and refractory cells, we hypothesized that JA may have exerted its effect *via* inhibition of the NF-κB pathway.

### JA directly bound to IKK to prevent the downstream activation of NF-B

**Figure 6:**
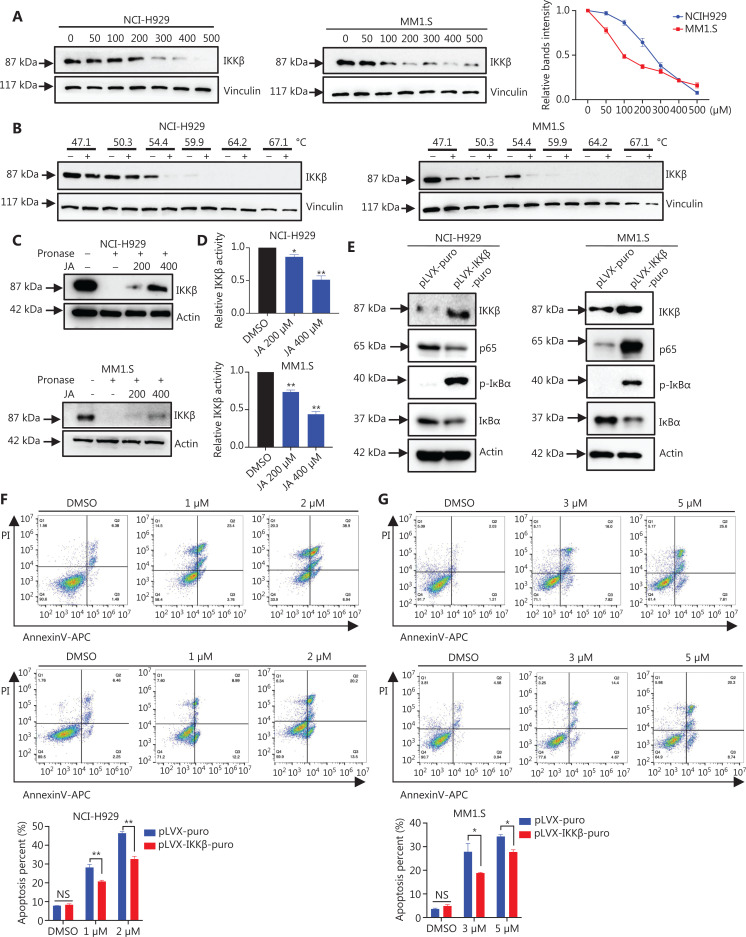
JaponiconeA (JA) exerted its effect by binding NF-κB IKKβ. (A, B) The thermal stabilization of IKKβ when incubated with JA using various dosages and temperatures. (C) The DARTS assay showed direct binding of JA to IKKβ. (D) IKKβ kinase activity was detected using a quantitative detection kit. (E) The overexpression of IKKβ in NCI-H929 or MM1.S cells and its effect on downstream targets of NF-κB were verified by Western blot. (F) MM cells transfected with IKKβ and control cells were treated with JA for 24 h, and cell apoptosis was determined by flow cytometry (*P < 0.05; **P < 0.01; *vs.* the control).

To identify the direct target of JA in the NF-κB pathway, an *in vitro* drug-target screening method using the cellular thermal shift assay (CETSA) was conducted^27^. Compared with the DMSO control, treatment with JA reduced the thermal stability of the IKKβ protein in NCI-H929 and MM1.S cells (**Figure 6A and 6B**). No alteration of p65 or IκBα was observed (**Supplementary Figure S4**). To determine if IKKβ was a direct target of JA, we conducted 3 types of biochemical experiments. Drug affinity responsive target stability (DARTS) assays were performed to show the direct binding between IKKβ protein and JA (**Figure 6C**), while *in vitro* IKKβ kinase activity assays were conducted to verify that JA competitively suppressed IKKβ kinase activity (**Figure 6D**). Moreover, to determine if JA exerted its effect by inhibiting IKKβ, we overexpressed IKKβ in MM cells. Remarkable increases of IKKβ, both in mRNA and protein levels, were observed after stable transfections with enhanced NF-κB-targeted genes (**Figure 6E, Supplementary Figure S5**). We then treated OE-IKKβ and control cells using the same dose of JA, which showed a significant decrease of apoptosis in the OE-IKKβ group when compared to that of the control group (**Figure 6F**). We also showed that knockdown of IκBα attenuated the apoptosis of JA (**Supplementary Figures S6 and S7**). Taken together, these results suggested that JA exerted anti-MM effects *via* inhibiting IKKβ to prevent activation of the NF-κB pathway.

## Discussion

The therapeutic effect and prognosis of myeloma have been significantly improved with the clinical use of proteasome inhibitors, immunomodulatory drugs, and monoclonal antibodies. However, due to evolving molecular changes, genetic mutations, and interactions with the BM microenvironment, patients with MM develop drug resistance, which remains a major clinical challenge. It is therefore critically important to identify novel agents to overcome drug resistance. The NF-κB signaling pathway was found to play a significant role in the promotion of MM and bortezomib resistance^11,28^. Moreover, inhibition of the NF-κB pathway overcame bortezomib resistance. Knockdown of USP7 significantly enhanced the sensitivity to bortezomib *via* stabilizing IκBα to inhibit the NF-κB pathway^13^. Inhibition of p65 activation by Ibrutinib or lenti-viral miRNA interference also restored sensitivity to bortezomib^12^. NEK2 contributes to bortezomib resistance through activation of NF-κB and destabilizing NEK2 kinase, which also helps to overcome resistance to proteasome inhibitors^29^. Furthermore, the epidermal growth factor receptor pathway substrate 8 (EPS8), a downstream target of NF-κB, was found to assist in the bortezomib resistance in MM cells^30^. Together, these studies suggested the possibility of overcoming bortezomib resistance by inhibition of the NF-κB pathway.

The lack of identifiable hydrophobic pockets in NF-κB transcription factor dimers makes it challenging to find an effective small molecule inhibitor. It could therefore be a promising strategy to perturb upstream factors that are essential for the activation of NF-κB, such as IKKβ. IKKβ is the logical, first-choice target for the development of pharmacological inhibitors of the NF-κB pathway^31^. IKKβ inhibitors have displayed significant therapeutic potential. For example, MLN-120B inhibited MM cell growth in a clinically SCID-hu mouse model^32^. In our study, we found another IKKβ inhibitor that inhibited the NF-κB pathway by directly interacting with IKKβ. When compared with MLN120B’s modest anti-MM activity, a lower dose of JA was able to exert a stronger anti-MM effect. Moreover, it might also be possible to identify more potent and selective IKKβ inhibitors, based on the chemical structure of JA.

For many years, natural products have been used to treat cancer; for example, harringtonine, camptothecin, paclitaxel, and bleomycin are still important treatments for malignancies. In addition, tubulin inhibitors such as vinflunine, curcumin, resveratrol, apigenin, and isothiocyanates have broad and promising clinical applications in cancer treatments^15^. Phytochemicals are still regarded as economical, accessible, readily applicable, and abundant sources for the identification of new drugs for cancer control and management^15^. However, one of the biggest challenges in drug research is to understand the underlying mechanisms of drug actions. To identify the direct targets, drugs should be chemically modified *in vitro* to capture the physically binding proteins, and surface plasmon resonance should be used to confirm the direct binding of drugs and targets, although it is usually time-consuming and costly. Moreover, due to the limitation of funds and technology, not every investigator can study the specific mechanism of action of drugs in this manner. We therefore screened the possible biological processes and pathways impacted by drugs of interest using biological information analysis. Using this methodology, we identified direct binding using CETSA, and DARTS, which were economical methods to verify the direct binding of drugs and their targets^27,33^. In addition, knockdown or overexpression targets were used to determine sensitivities to drugs, and further characterize the relationships between drugs and their targets. These strategies can provide economical and convenient preliminary methods to characterize the mechanism of action of drugs.

In summary, we showed that JA had potent and selective anti-myeloma activities. It induced cell apoptosis and G2/M phase arrest, which partially overcame bortezomib resistance *via* inactivation with IKKβ. Considering that IKKβ is the most abundant and important IKK in MM, inhibition of IKKβ by JA is a promising strategy to inhibit the NF-κB pathway in MM, suggesting that JA may be an effective treatment for MM patients.

## Conclusions

**Figure 7:**
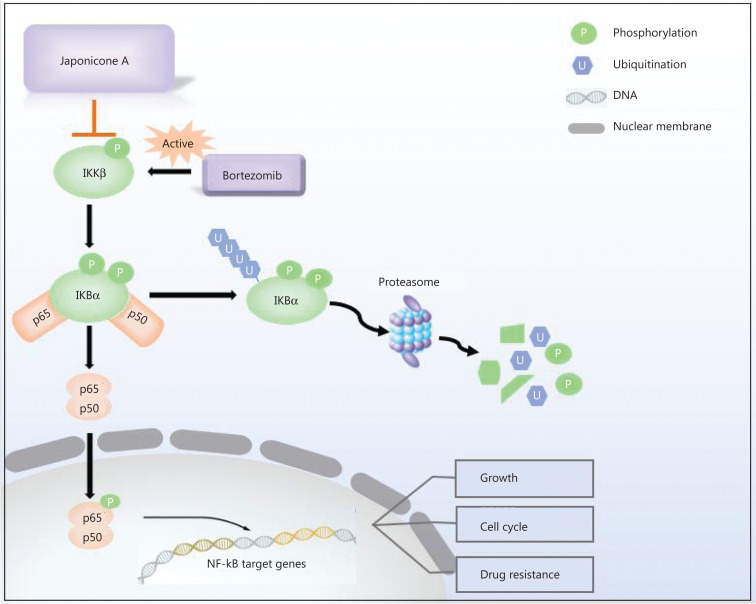
Mechanisms of the effects of JaponiconeA (JA) on MM cells. Bortezomib activates NF-κB IKKβ, which subsequently phosphorylates IκBα. After proteasome degradation of IκBα, p50/p65 translocate to the nucleus to exert their functions. JA blocks the phosphorylation of IKKβ, and suppresses the IKKβ-IκBα-NF-κB axis, which enhances bortezomib-induced cytotoxicity.

Our findings showed that JA exhibited strong anti-tumor effects in MM cells. It sensitized myeloma cells to bortezomib and overcame NF-κB-induced drug resistance by inhibiting IKKβ (**Figure 7**), thus providing a possible novel treatment for MM patients.
