# Supplementary material for: JaponiconeA induces apoptosis of bortezomib-sensitive and -resistant myeloma cells in vitro and in vivo by targeting IKKβ
Source: Cancer Biol Med. 2021 Sep 28;19(5):651–68. doi: 10.20892/j.issn.2095-3941.2020.0473 (PMC9196056; doi:10.20892/j.issn.2095-3941.2020.0473)
Supplement: Supplementary file 1 [file cbm-19-651-s001.pdf]

Supplementary materials

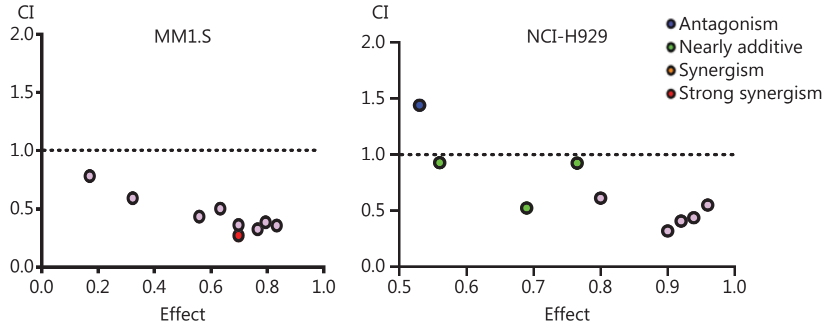

Figure S1 MM cells were treated with bortezomib and JaponiconeA, separately or together for 24 h, and the inhibition was detected using the CCK8 assay. The combination index (*CI*) was then analyzed using CompuSyn software.

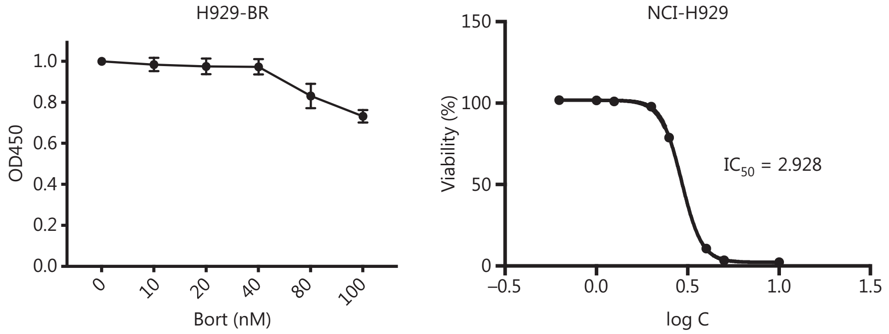

Figure S2 H929-BR or NCI-H929 cells were treated with bortezomib for 24 h, and the IC_{50} was calculated using GraphPad prism software.

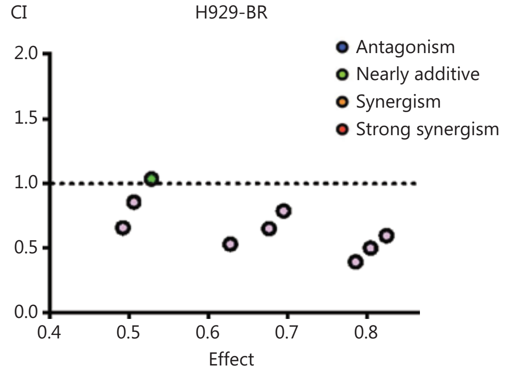

Figure S3 H929-BR cells were treated with bortezomib and JaponiconeA, separately or together, and the inhibition was detected using the CCK8 assay. The combination index (*CI*) was analyzed using CompuSyn software.

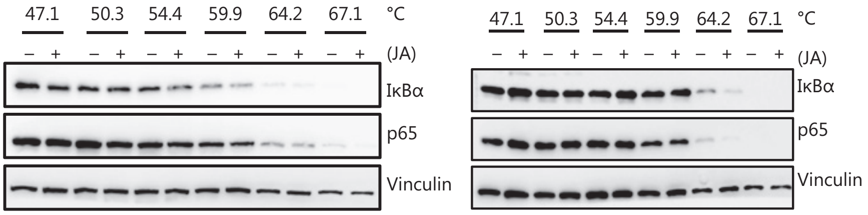

Figure S4 After MM cells were incubated with JaponiconeA at various temperatures, the thermal stabilizations of IκBα and p65 were detected by Western blot.

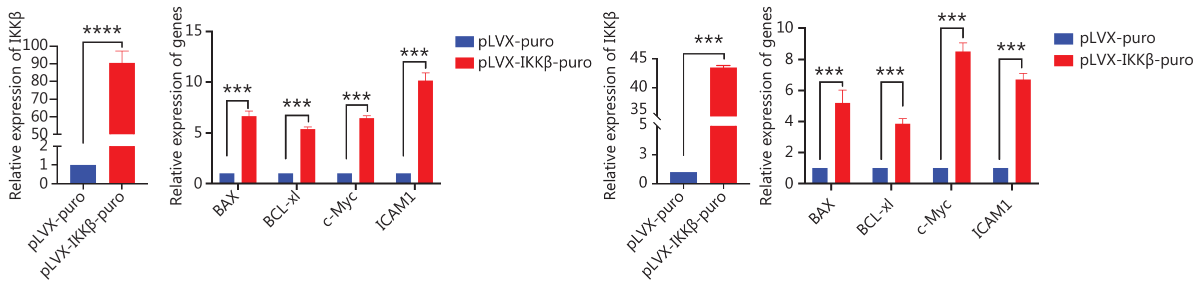

Figure S5 The mRNA level of NF-κB IKKβ was detected in IKKβ-overexpressed NCI-H929 or MM1.S cells, and the mRNA expression of downstream targets of NF-κB were detected using q-PCR (***P < 0.001; ****P < 0.0001 *vs.* the control).

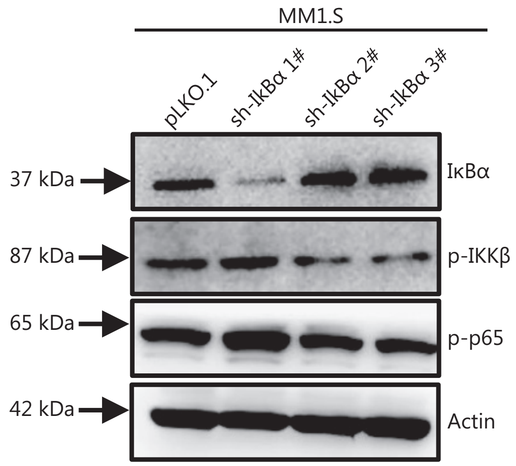

Figure S6 The knockdown of IκBα in MM1.S cells, and its downstream effect on NF-κB was determined using Western blot.

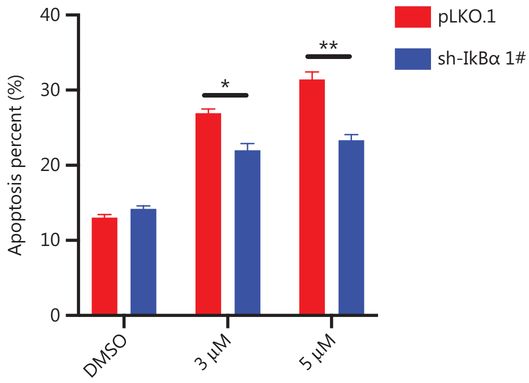

Figure S7 MM1.S cells transfected with sh-IκBα 1# and control cells were treated with JA for 24 h, then cell apoptosis was determined using flow cytometry (*P < 0.05; **P < 0.01).
